# Immunotherapy of COVID-19 with Bacille Calmette – Guerin: Where is the missing red herring?

**DOI:** 10.4102/sajid.v36i1.215

**Published:** 2021-03-05

**Authors:** Hari M. Saxena

**Affiliations:** 1 Department of Veterinary Microbiology, Guru Angad Dev Veterinary and Animal Sciences University, Ludhiana, India

**Keywords:** BCG, COVID 19, PPD, SARS CoV2, coronavirus, immunotherapy, vaccine

## Abstract

Coronavirus Disease 2019 (COVID-19) morbidity and mortality was found to be less severe in countries where Bacille Calmette – Guerin (BCG) vaccination of the population is carried out. Conjugating Purified Protein Derivative (PPD) onto tumour cells and injecting into BCG primed mice was found to enhance anti-tumour immune response. We had proposed earlier that *in vitro* activated autologous anti-tumour T-cells bearing Major Histocompatibility Complex (MHC) II on their surface, if pulsed with PPD and re-infused in a BCG – primed patient, can activate PPD – specific helper T-cells and the focused secretion of lymphokines like the IL-2 can selectively amplify the antitumor T-cell response by their proliferation and activation in a specific manner bypassing the suppression exerted by the anti-idiotypic and suppressor cells. The prime – boost strategy with the BCG–PPD system can also be applied to the immunoprophylaxis and immunotherapy of COVID-19. The autologous anti-Corona virus B and T lymphocytes can be activated *in vitro* by inactivated virus or mitogens like Concanavalin A to express MHC class II molecules on their surface and pulsed with PPD for carrier targeting *in vivo*. Such PPD – pulsed activated (MHC-II+ve) anti-viral lymphocytes if transfused back into a patient already vaccinated with BCG during childhood or primed with BCG during adulthood 2 weeks before transfusion, could lead to a high magnitude of selective *in vivo* amplification of specific anti-viral lymphocytes, which can mount adequate and appropriate immune response to get rid of the virus and cure the patient from COVID-19. Conjugating antigens to PPD and injecting into BCG primed humans may also be helpful for immunoprophylaxis against COVID-19. Thus, PPD may prove to be the red herring in the BCG therapy of COVID-19.

## Introduction

Bacille Calmette – Guerin (BCG), the live attenuated mycobacterial vaccine has been in use against tuberculosis for over a century and is still being used in several countries. The BCG vaccine is given to more than 130 million babies worldwide each year to protect against tuberculosis.^1^ Besides its use as a vaccine, BCG has also been used as an immunostimulant in some other infectious diseases and cancer. It has recently been reported that coronavirus disease 2019 (COVID-19) morbidity and mortality has been less severe in certain countries where childhood vaccination of the population with BCG is commonly practiced.^2^ The notable examples are India and some African countries. In multiple studies comparing the effect of COVID-19 worldwide, findings thus far suggest lower rates of infection and mortality in countries with universal neonatal BCG tuberculosis vaccination policies compared with countries without this practice.^3^ In another study, it was found that every 10% increase in the BCG index (which reflects the extent of a country’s BCG vaccination) was associated with a 10.4% reduction in mortality from COVID-19, and higher mortality rates were observed in countries with later versus early initiation of universal BCG vaccination.^4^

However, BCG alone may not be the ultimate immunotherapy of choice for COVID-19 because it causes generalised non-specific immunostimulation where anti-idiotypic and suppressor lymphocytes may also proliferate dampening the specific anti-viral immune response against the SARS CoV2 virus because of immunoregulation. Furthermore, the harmful cytokines released by the suppressor cells, the phagocytes and other cells under the influence of BCG may contribute to the cytokine storm elicited because of the Corona virus infection. These undesirable and harmful effects of the non-specific immunostimulation by the BCG can be mitigated if the specific anti-viral immunity can be scaled up by focusing the lymphokine release by the helper T-lymphocytes onto the anti-viral lymphocytes (both B- and T-cells) of the patient.

## Mechanism of action of Bacille Calmette – Guerin and purified protein derivative

When exposed to *Mycobacterium* tuberculosis antigen, the sensitisation initiates in the regional lymph nodes where T-lymphocytes proliferate in response to the antigenic stimulus to give rise to specifically sensitised lymphocytes, which may exist in the circulation up to many years. Antigen is presented to T-cells by being ingested by antigen presenting cells (APC), which then present it on their surface to lymphocytes in combination with various major histocompatibility molecules once they reach local lymph nodes. Purified protein derivative (PPD) is a source of mycobacterial antigens, which consist of approximately 200 protein allergens obtained from the precipitate of *M. tuberculosis* culture supernatant. When these antigens are injected intradermally into the skin, activated T-lymphocytes mount immune response to these antigens. Molecular analyses of PPD has revealed that four heat shock proteins (GroEl, GroEs, DnaK and HspX) contribute to roughly 60% of the PPD proteomic content. These chaperone proteins share a high homology and are conserved amongst most mycobacterial species.^5,6^

Purified protein derivative most likely interacts with toll-like receptor 2 expressed on APCs that initiates an inflammatory response. Subsequent restimulation of the sensitised lymphocytes with the same or a similar antigen, such as the intradermal injection of PPD, evokes a local reaction mediated by these cells. This reaction is referred to as a delayed-type hypersensitivity response that includes vasodilation, edema and the infiltration of lymphocytes, basophils, monocytes and neutrophils into the site of antigen injection. The sensitised antigen-specific T-lymphocytes proliferate and release lymphokines, which mediate the accumulation of other cells at the site. *In vitro* studies show that PPD promotes the upregulation of vascular endothelial growth factor (VEGF) expression in T-lymphocytes through MHC class II interaction with CD4+ T-lymphocyte interaction.^7^ The reactions are evident after 5–6 h following administration.

Purified protein derivative is a cell-free purified protein fraction obtained from a human strain of *M. tuberculosis* grown on a protein-free synthetic medium and inactivated. The sensitisation following BCG injection occurs primarily in the regional lymph nodes. T-lymphocytes proliferate in response to the antigenic stimulus to give rise to specifically sensitised lymphocytes. After 3–8 weeks, these lymphocytes enter the blood stream and circulate for years. Subsequent restimulation of these sensitised lymphocytes with the PPD evokes a strong cellular immune response.

Purified protein derivative is a complex mixture of mycobacterial peptides, which cannot initiate an immune response to itself when injected alone in the body of a naïve animal or human not sensitised with mycobacterial antigens. However, if PPD is injected in an animal presensitised with mycobacterial antigens, it elicits a strong immune response to itself. Purified protein derivative acts as a strong carrier molecule when conjugated with a hapten and injected in an animal presensitised with mycobacterial antigens,^8^ for example, infection with mycobacteria or vaccination with BCG. It stimulates T-helper response to itself and a high magnitude immune response (humoral and cellular) to the hapten conjugated to it because of the linked recognition of the hapten and the carrier PPD. The same principle has been exploited earlier to enhance anti-tumour immune response^9^ by conjugating PPD onto the tumour cells and injecting it into BCG primed mice. However, in this model, the lymphokine secretion by PPD – specific helper T-cells will be directed onto the target PPD – bearing tumour cells and the proliferation of anti-tumour lymphocytes will be the result of the bystander effect.

## The hypothesis of strategy for selective *in vivo* immunostimulation

An alternative strategy proposed by us^10^ envisaged a focused secretion of lymphokines like IL2 by helper T-cells directly onto the effector lymphocytes (anti-tumour T-cells in our case) bringing in a high magnitude of selective *in vivo* amplification of desired T-cells minimising the chances of non-specific amplification of harmful lymphocytes like the anti-idiotypic or the suppressor lymphocytes and phagocytes.

B-cells constitutively express MHC class II molecules and can process and present antigens to helper T-cells. Activated T-lymphocytes (including the cytotoxic T-cells) from some species of animals such as rats and humans express MHC class II molecules on their surface and can present antigenic peptides to the helper T-cells. Purified protein derivative is a complex mixture of mycobacterial peptides and can be presented by MHC II bearing cells without any further processing.

**FIGURE 1:**
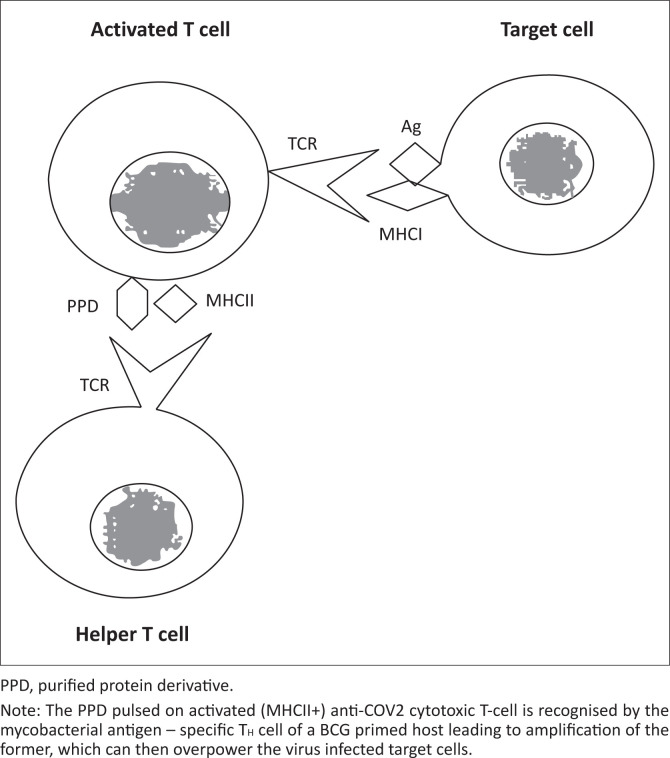
Diagrammatic representation of purified protein derivative mediated selective *in vivo* amplification of a T-cell.

We had proposed^10^ that *in vitro* activated autologous anti-tumour T-cells bearing MHC II on their surface, if pulsed with PPD and re-infused in a BCG – primed patient, can activate PPD – specific helper T-cells and the focused secretion of lymphokines like the IL-2 can selectively amplify the anti-tumour T-cell response by their proliferation and activation in a specific manner bypassing the suppression exerted by the anti-idiotypic and suppressor cells (Figure 1).

Bacille Calmette – Guerin boosts the immune system to fight not only against tuberculosis but other infections also. A 2018 study suggested that the BCG vaccine reduced yellow fever vaccine viremia by 71% in volunteers in the Netherlands. Two mice studies showed that it reduces the severity of mengovirus (encephalomyocarditis virus) infection.^1^ Purified protein derivative immunotherapy for the treatment of warts involves single or multiple intradermal injections of PPD antigens in the substance of viral warts at the specific interval till there is a disappearance of viral warts. Repeated injections mount booster response to PPD antigens. Activated T-lymphocytes release gamma interferons at the site of intradermal PPD injections, thereby killing viral particles of human papillomavirus (HPV). It has also been observed in various studies that distant noninjected viral warts at the site away from PPD injections also respond to the therapy indicating that the release of interferon gamma is not just local but a systemic response.^11^

## The missing red herring in Bacille Calmette – Guerin immunotherapy of coronavirus disease 2019

The prime – boost strategy with the BCG–PPD System described here can also be applied to immunoprophylaxis and immunotherapy of several infectious diseases including COVID-19. The autologous anti-Corona virus B- and T-lymphocytes can be activated *in vitro* by inactivated virus or mitogens such as Concanavalin A to express MHC class II molecules on their surface and pulsed with PPD for carrier targeting *in vivo*. Such PPD – pulsed activated (MHC-II+ve) anti-viral lymphocytes if transfused back into a patient already vaccinated with BCG during childhood or primed with BCG during adulthood 2 weeks before transfusion, could lead to a high magnitude of selective *in vivo* amplification of specific anti-viral lymphocytes, which can mount adequate and appropriate immune response to get rid of the virus and cure the patient from COVID-19.

## Conclusion

The inactivated SARS CoV2 virus, or its subunit fractions such as the spike proteins, synthetic peptides or new recombinant vaccines against the Corona virus can be made highly immunogenic by conjugating the antigen to PPD and injecting it into BCG vaccinated or BCG primed individuals for immunoprophylaxis. The hypothesis can be tested in an appropriate animal model employing an inactivated animal Corona virus as the vaccine candidate. The lymphokines secreted by such prime – boost immunisation may be conducive to generation of long – lived memory cells granting long-term immunity against the Corona virus. The strong carrier effect induced by linking PPD to SARS COV2 antigen and injecting in a BCG primed patient can lead to a high magnitude of selective *in vivo* amplification of anti-Corona virus B and T lymphocytes capable of overpowering the pathogen. Thus, PPD could prove to be the red herring in the control of COVID-19.
